# A combination of single-balloon enteroscopy-assisted laparoscopy and endoscopic mucosal resection for treating gastrointestinal venous malformations in blue rubber bleb nevus syndrome: a case report

**DOI:** 10.1186/s12876-020-01328-6

**Published:** 2020-06-10

**Authors:** Yuemei Xu, Yingjie Wu, Zhenzhen Dai, Feizhen Xia, Feng Xu

**Affiliations:** Department of Gastroenterology, Yinzhou Hospital affiliated to Medicine School of Ningbo University, 251 Baizhang Rd. Ningbo, Zhejiang, 315040 China

**Keywords:** Blue rubber bleb nevus syndrome, Endoscopic mucosal resection, Laparoscopy, Single-balloon endoscopy, Gastrointestinal

## Abstract

**Background:**

Blue rubber bleb nevus syndrome (BRBNS) is a rare congenital disease characterized by multifocal venous malformations. It remains a considerable challenge in treating the gastrointestinal (GI) venous malformations due to multiple lesions throughout the GI tract, and the likelihood of recurrence. We report a case study of BRBNS in the GI tract, in which GI venous malformations and related GI bleeding were successfully treated with a combination of multiple endoscopic procedures.

**Case presentation:**

A 17-year-old man was admitted to our hospital for dizziness and hypodynamia. The symptoms persisted for nearly 1 year. The laboratory tests revealed iron-deficiency anemia with abnormally low hemoglobin (Hb), and a strong positive fecal occult blood test. A total of four hemangiomas were detected: one in the stomach, one in the descending colon, and two in the small intestines with a high risk of hemorrhage. Under gastroendoscopy, enteroscopy, and video capsule endoscopy (VCE) throughout the GI tract, the patient underwent surgical treatment. Endoscopic mucosal resection was initially performed in the stomach and colon, and the lesions in the small intestine were resected with laparoscopy auxiliaried by single-balloon enteroscopy (SBE), during which SBE assisted in identifying the lesions. The patient well-tolerated the procedures, and had a favorable prognosis.

**Conclusion:**

The combination of single-balloon enteroscopy-assisted laparoscopy and endoscopic mucosal resection was effective for the present case, which could be considered for patients with similar clinical conditions.

## Background

Blue rubber bleb nevus syndrome (BRBNS) is a rare congenital disease, in which malformed veins appear on the skin, and on the surface of internal organs. Since BRBNS was first described in 1958 by Bean, approximately 200 cases have been reported. This sporadically occurs, but has been postulated with dominant autosomal inheritance and confirmed with a linkage of familial forms of venous malformation to chromosome 9p [1]. BRBNS is featured by multiple skin lesions and gastrointestinal (GI) venous malformations. Serious GI hemorrhage [2] is frequently observed in BRBNS patients, who also develop anemia, and require blood transfusions and lifelong treatment with iron supplement. The treatment of multiple lesions of the GI tract has remained very challenging, which was mainly due to the large number of lesions throughout the GI tract. Thus, there is a need to find more effective treatments for GI tract venous malformations and the eradication of GI bleeding.

We describe a case of BRBNS with multiple lesions throughout the GI tract, which was successfully treated with a combination of single-balloon enteroscopy-assisted laparoscopy and endoscopic mucosal resection (EMR).

## Case presentation

### Patient description and clinical examinations

A 17-year-old male patient was admitted to our hospital due to dizziness and hypodynamia. The symptom persisted for nearly 1 year. The physical examination identified a dark blue, soft lobulated lesion (5 mm in size) on the patient’s shoulder. The laboratory tests revealed iron-deficiency anemia with abnormally low hemoglobin (Hb, 4.7 g/dl), and a strong positive fecal occult blood test.

**Fig. 1:**
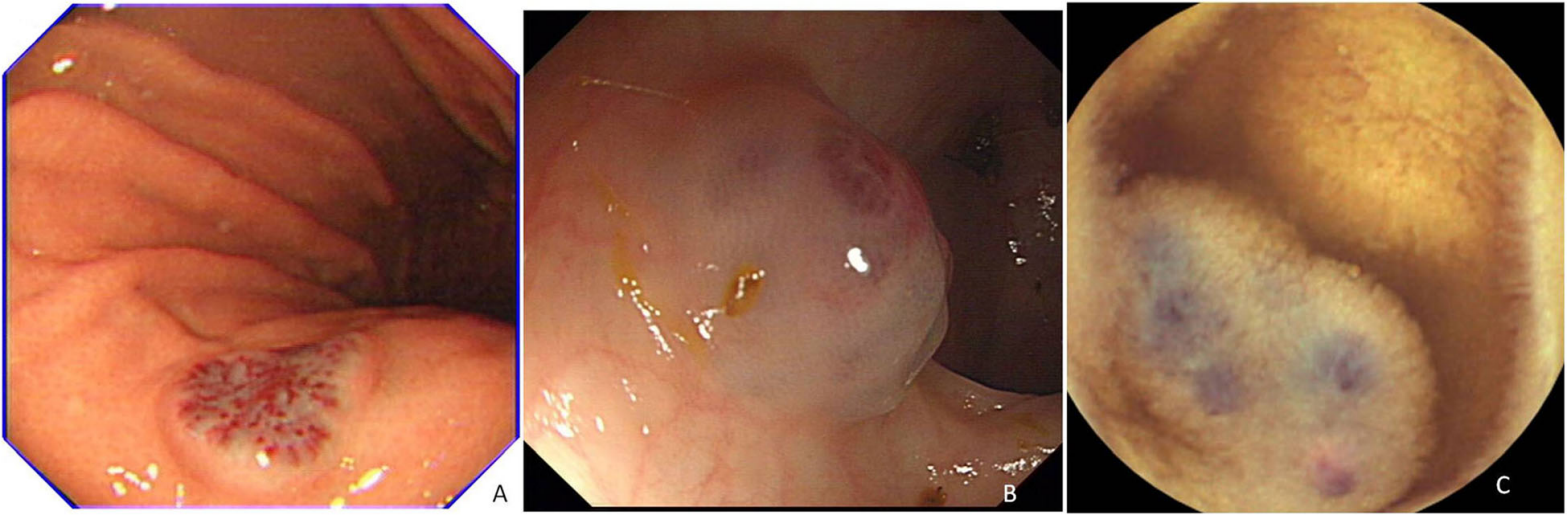
The hemangiomas in the GI tract as detected by endoscopic examinations. **a** A hemangioma in the lesser curvature of the stomach without any stigmata of the recent hemorrhage; **b** A hemangioma in the descending colon; **c** Pink-bluish pedunculated and sessile venous malformations in the small intestine found by video capsule endoscopy

The patient received blood transfusion, and the symptoms improved. Further diagnostic endoscopies were conducted for the patient. The gastroendoscopy revealed a hemangioma of approximately 1.0 cm in diameter and a lesion in the lesser curvature of the stomach without any stigmata of the recent hemorrhage (Fig. 1-A). The colonscopy detected a hemangioma in the descending colon (Fig. 1-B). Video capsule endoscopy (VCE) was performed, and two pink-bluish pedunculated and sessile venous malformations were identified in the small intestine of approximately 1.0 cm in diameter (Fig. 1-C). A total of four hemangiomas were detected from the patient: one in the stomach, one in the descending colon, and two in the small intestine with a high risk of hemorrhage. The study was reviewed and approved by the Ethics Committee of the Yinzhou Hospital Affiliated to Ningbo University. A written informed consent was obtained from the patient’s parent.

### Final diagnosis

The final diagnosis of the presented case was BRBNS with multiple lesions throughout the GI tract.

### Treatment

**Fig. 2:**
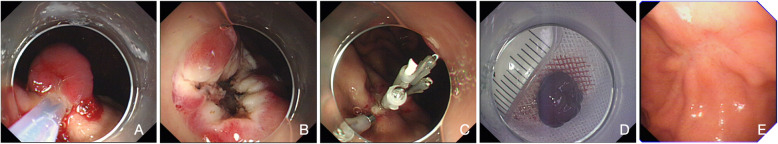
Intraoperative images of the lesions in the stomach. **a** The lesion in the stomach using endoscopic mucosal resection; **b** The surface of the wound; **c** Closing of the wound using clips; **d** The specimens taken from the lesion; **e** The gastroscopy revealed the scar of the lesion in the stomach

**Fig. 3:**
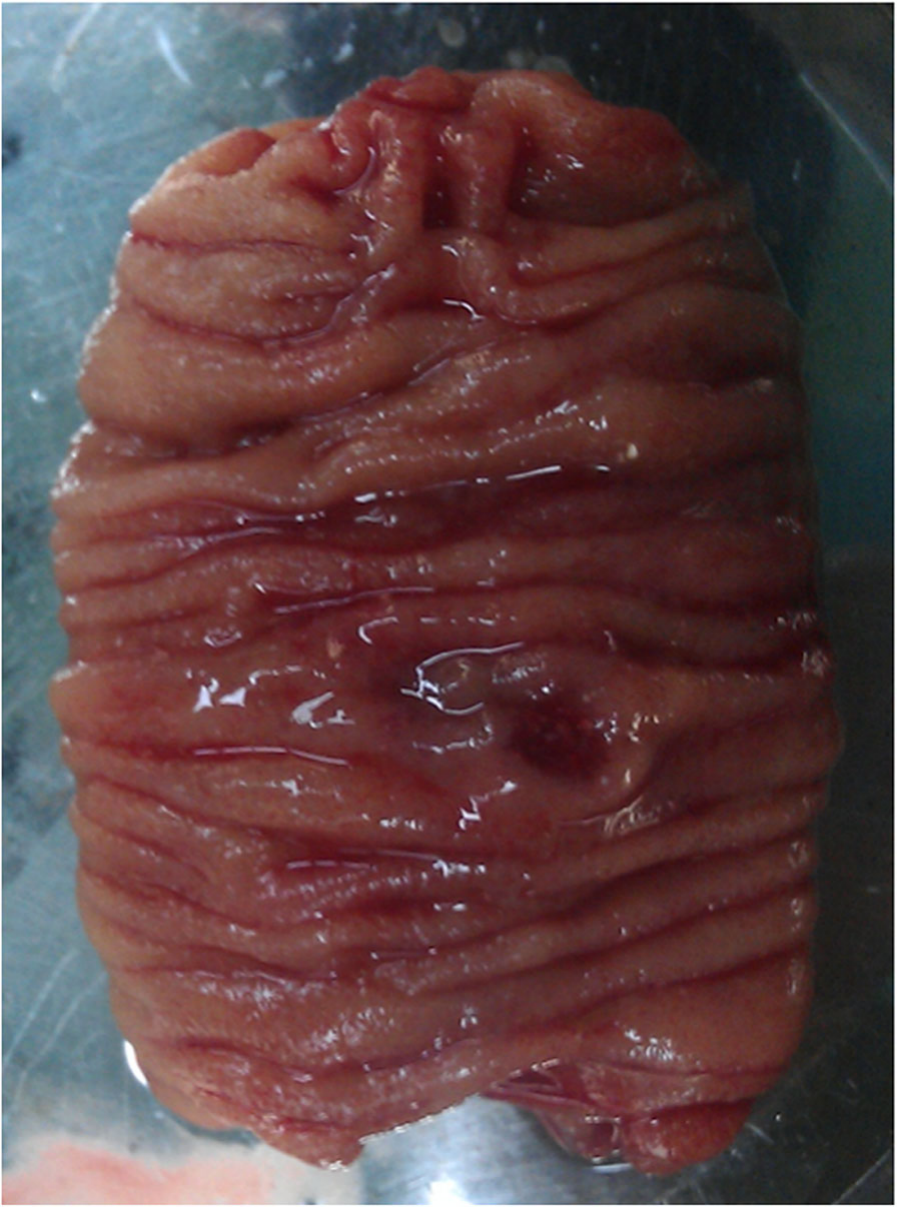
Intraoperative image of the lesion in the small intestine. The specimen taken from the lesion in the small intestine during the surgical segmental resection

**Fig. 4:**
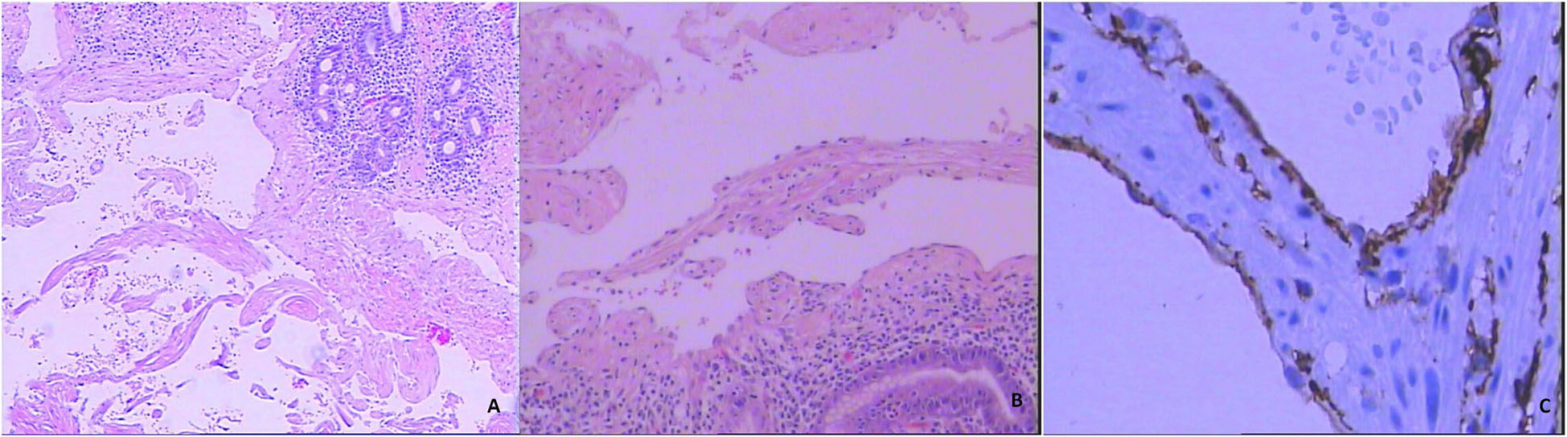
Histopathologic findings of the lesions. **a** The hematoxylin and eosin (H&E) staining of blood-filled ectatic vessels (5 × 10); **b** The H&E staining of blood-filled ectatic vessels (10 × 10); **c** The endothelial cells surrounded by thin connective tissues that were CD34 positive (40 × 10)

The patient received blood transfusion, and the symptoms improved. In order to surgically remove the GI lesions and minimize procedure-related adverse events (such as bleeding), single-balloon enteroscopy-assisted laparoscopy combined with EMR was performed, during which all four GI lesions were successfully removed. The surgery was performed in two sessions. First, the EMR was performed in the stomach and colon, during which 0.9% saline with indigo was injected to the submucous, the lesion was cut with snare, and the wound was closed with clips (Fig. 2a, b, c and d). Second, the lesions in the small intestine were resected with laparoscopy auxiliaried by SBE, during which SBE assisted in identifying the lesion (Fig. 3). The histopathologic examination of the lesions revealed blood-filled ectatic vessels lined by a single layer of endothelial cells surrounded by thin connective tissues that were CD34 positive (Fig. 4a, b and c). Based on the clinical presentation, endoscopic findings and postoperative histopathological examinations, the final diagnosis of BRBNS was made and confirmed in the patient.

### Outcome and follow-up

The patient recovered well from the surgical treatment, with a normal GI passage at 3 days following the surgery. The patient was discharged from the hospital after 4 days and prescribed with iron supplement. Fortunately, during the six-month follow-up visits, the patient had neither occult blood in his feces, nor anemia. The endoscopy examination of the stomach and colon revealed no residue or recurrence (Fig. 2-E).

## Discussion and conclusion

BRBNS is a rare vascular disorder, and patients with BRBNS present with bluish venous malformations that most commonly affect the skin and GI tract, and occasionally affect other internal tissues (e.g. the liver, spleen, kidney, brain and joints). In addition, previous studies have shown some very uncommonly involved tissues. For example, Aroor et al. described a case of BRBNS with an atrial septal defect [3]. Huang et al. reported a case of BRBNS with both GI and central nervous system involvement. We report a BRBNS case, in which both skin and GI venous malformations were present.

GI venous malformations often bleed, and massive or occult GI hemorrhage with iron-deficiency anemia is a typical clinical feature [4]. Other clinical manifestations include abdominal pain, chronic consumption coagulopathy, intestinal intussception, and infarction. Esposito et al. reported that intestinal intussusception was present in a BRBNS patient [1]. In the present case, the patient complained of dizziness and hypodynamia as a consequence of hemorrhage and anemia.

It has been noted that GI lesions in BRBNS can vary in location and number, and forms of lesions. The most common site in the GI tract is in the small intestine (100%), followed by the colon (74%) and stomach (26%) [5]. Endoscopy has been considered as the most reliable evaluation for lesions in the GI tract, which include VCE [6], single-balloon enteroscopy, double-balloon enteroscopy [7], gastroscopy and colonoscopy. GI BRBNS can manifest in diverse forms, including polylobulated, nodular, sessile, pedunculated, or ulcerated. In the present case, gastroscopy, colonoscopy and VCE were performed to examine the lesions in the whole gut, and four lesions were identified. In order to minimize procedure-related complications, single-balloon enteroscopy (for the orientation of the incision for small intestine lesions)-assisted laparoscopy coupled with endoscopic mucosal resection was applied to remove all four GI lesions. These procedures appeared to be safe and effective for the patient. The management of BRBNS remains considerably challenging, and the lesions in the skin rarely bleed. In contrast, GI lesions manifest throughout the GI tract and begin to bleed at an early age, persisting for the rest of a person’s life [3]. The treatment plan is dependent on the extent of the GI involvement and severity of the clinical condition. Conservative treatment is effective for small bleeding. However, resections are required to eradicate large GI bleeding [8]. It has been reported that a number of treatment (e.g. polidocanol foam sclerotherapy [9], cryosurgery and argon plasma coagulator) combinations were more effective, and led to a better prognosis for patients with GI BRBNS, when compared with single treatment alone. During the establishment of the treatment plan for the present patient, the age, chronic iron deficiency anemia, and locations of the four lesions in the GI tract were considered for the patient. EMR was performed to treat both lesions in the stomach and colon, and this was able to completely excise the malformation without bleeding. It is noteworthy that polypectomy was not used for the lesions in the small intestine mainly due to the shortage of equipment. During the six-month follow-up visit, no recurrence of GI lesions occurred in the patient.

In summary, the findings of the present case study suggest that venous malformations throughout the GI tract can be successfully excised by polypectomy, regardless of the number and size of lesions. Furthermore, the combination of single-balloon laparoscopy and EMR was effective for the present case, and can be considered for patients with similar clinical conditions.

## Data Availability

The data of this study are available from the corresponding author upon reasonable request.

## Abbreviations

BRBNS: Blue rubber bleb nevus syndrome
GI: Gastrointestinal
Hb: Hemoglobin
VCE: Video capsule endoscopy
